# 

**Published:** 1897-07

**Authors:** 


### CONTENTS:

### SELECTIONS.

Article I.—Care of the Mouth in Relation to Prophylaxis of the Digestive Tract. By G. V. E. Brown.......... 97
" II.—Ambidexterity. By Herbert Lake. L. D. S........... 104
" III.—How Can We Educate the Public? By Dr. B. J. De-Vries.......... 108
" IV.—Dental Hygiene. By J. Clifford Wing.......... 109
" V.—Light in Vacuum Tubes.......... 112
" VI.—Varnishing Cavities. By Dr. W. G. Browne.......... 114
" VII.—An Apparatus for Drainage. By Dr. M. L. Rhein.... 115
" VIII.—Listerin. By Dr. W. C Barrett.......... 118
" IX.—Filling Roots. By Dr. R. C. Young.......... 119
" X.—Dental Laws and Their Enforcement. By Dr. A. M. Callahan.......... 121
" XI.—Three Pathological Cases. By I. P. Wilson, D.D.S... 126
" XII.—Soldering.......... 130
" XIII.—Cataphoresis in the Removal of Vital Pulps followed by Immediate Root Filling. S. Eldred Gilbert, D.D.S. 135

### OBITUARY.

Resolutions in Memory of Dr. Frank Abbott.......... 137

### MONTHLY SUMMARY.

New Method of Administering Chloroform.......... 138

Pearson's Local Anæsthetic.......... 138

Pulp Devitalization in the Teeth of Children.......... 139

A Litter of Supernumary Teeth.......... 139

Metal Pins.......... 140

Treating Pulpless Teeth.......... 141

Catching Cold.......... 142

The Sense of Touch.......... 143

The Antiseptic Properties of Saliva.......... 143

A Useful Flux.......... 144

